# Construction and Mechanism Exploration of Highly Efficient System for Bacterial Ghosts Preparation Based on Engineered Phage ID52 Lysis Protein E

**DOI:** 10.3390/vaccines12050472

**Published:** 2024-04-28

**Authors:** Yi Ma, Sijia Wang, Bin Hong, Lan Feng, Jufang Wang

**Affiliations:** School of Biology and Biological Engineering, South China University of Technology, Guangzhou 510006, Chinajufwang@scut.edu.cn (J.W.)

**Keywords:** bacterial ghosts, *Escherichia coli* phage ID52, lysis protein E, *Escherichia coli* Nissle 1917, *Salmonella* pullorum, *Salmonella* choleraesuis

## Abstract

Bacterial ghosts (BGs) are hollow bacterial cell envelopes with intact cellular structures, presenting as promising candidates for various biotechnological and biomedical applications. However, the yield and productivity of BGs have encountered limitations, hindering their large-scale preparation and multi-faceted applications of BGs. Further optimization of BGs is needed for the commercial application of BG technology. In this study, we screened out the most effective lysis protein ID52-E-W4A among 13 mutants based on phage ID52 lysis protein E and optimized the liquid culture medium for preparing *Escherichia coli* Nissle 1917 (EcN). The results revealed a significantly higher lysis rate of ID52-E-W4A compared to that of ID52-E in the 2xYT medium. Furthermore, EcN BGs were cultivated in a fermenter, achieving an initial OD_600_ as high as 6.0 after optimization, indicating enhanced BG production. Moreover, the yield of ID52-E-W4A-induced BGs reached 67.0%, contrasting with only a 3.1% yield from φX174-E-induced BGs. The extended applicability of the lysis protein ID52-E-W4A was demonstrated through the preparation of *Salmonella* pullorum ghosts and *Salmonella* choleraesuis ghosts. Knocking out the molecular chaperone gene *slyD* and *dnaJ* revealed that ID52-mediated BGs could still undergo lysis. Conversely, overexpression of integral membrane enzyme gene *mraY* resulted in the loss of lysis activity for ID52-E, suggesting that the lysis protein ID52-E may no longer rely on SlyD or DnaJ to function, with MraY potentially being the target of ID52-E. This study introduces a novel approach utilizing ID52-E-W4A for recombinant expression, accelerating the BG formation and thereby enhancing BG yield and productivity.

## 1. Introduction

BGs represent non-viable bacterial structures derived from Gram-negative bacteria. Despite lacking cytoplasmic contents, BGs retain the structural integrity of bacterial membranes, preserving surface antigens recognized by the immune system [1,2]. Consequently, when BGs enter the human or animal body, they can trigger the immune system to produce the required antibodies [3]. Renowned for their high load-bearing capacity, composite preservation, stability at room temperature, scalability, and adaptability, BGs find application across diverse domains. They serve as promising vaccine candidates [3,4,5], as drug delivery carriers [6,7], to present antigens [8], and as immune stimulants to induce inflammatory response [9]. Moreover, BGs possess innate immunostimulatory properties, eliminating the need for additional adjuvants and holding potential as natural adjuvants for future vaccine formulations and cancer treatments [10,11].

BGs can be generated through various methods, including genetic [12], chemical [13], phage lysis [14], or antimicrobial peptide treatment methods [15]. Among these, genetic engineering stands as the most commonly employed approach. Chemical methods, while feasible, may induce significant damage to cell wall antigens, potentially compromising the antigenicity and immunogenicity of BGs [16,17]. Despite the manifold advantages and broad application of BGs across diverse fields, the persistent challenge of low BG yield and productivity remains unresolved. Traditionally, BGs have been primarily prepared by tightly regulating the recombinant expression of the *E. coli* phage φX174 lysis gene E. Lubitz et al. discovered that phage φX174 encodes a single lysis gene, E, whose function is instrumental in inducing lysis in Escherichia coli, and showed that transmembrane tunnels penetrating both the inner and outer membranes are formed during lysis of protein E, directly visualizing the transmembrane lysis structure [18]. In addition, they observed that the specialized oligomeric structures that penetrate the inner and outer membranes of *Escherichia coli* are formed during the lysis of the E protein [19]. Fu et al. [20] mutated the temperature-sensitive promoter, enhancing thermal stability, albeit at the expense of lysis efficiency and BG formation. Conversely, Ma Yi et al. [21] achieved an initial ghost lysis induction OD_600_ of 2.0 by jointly controlling the T7 promoter and pLysS plasmid. However, the inducer isopropyl β-D-1-thiogalactopyranoside (IPTG) exhibits specific cytotoxic properties [22] and is cost-prohibitive, limiting its large-scale production and application in BGs. As an alternative, some researchers have recombinantly expressed the *E. coli* phage ID52 lysis protein ID52-E, utilizing an L-arabinose inducible promoter (araC-ParaBAD) to enhance the lysis effect of ID52-E [23], achieving an induction OD_600_ as high as 2.5.

The remarkable product safety exhibited by *E.coli* Nissle 1917 (EcN) positions it as a highly promising microbial chassis for the synthesis of metabolites and proteins with both medical and industrial applications [24]. Several studies have consistently highlighted the safety and efficacy of utilizing EcN ghosts as carriers for chemotherapy drugs in disease treatment, showcasing their enhanced tumor-killing potential compared to chemotherapy alone [25,26]. Beyond probiotics such as EcN, recent attention has focused on pathogenic bacteria in animal vaccines. *Salmonella enterica* subsp. *enterica* serovar pullorum (SP) is a pathogenic bacterium causing poultry septicemia leading to pullorum, an acute systemic disease associated with high mortality and persistent infection in embryos and chicks [27,28]. Similarly, *Salmonella enterica* subsp. *enterica* serovar choleraesuis (SC), a significant *Salmonella* strain, is a food-borne pathogen triggering systemic diseases accompanied by sepsis. It not only impairs the pig’s digestive system but also diminishes production performance or even causes death [29]. Diseases transmitted vertically by *Salmonella* continue to pose a substantial threat to both humans and animals [30], constituting a global public health concern. A number of *Salmonella* vaccines are available, including 9R Strain of *Salmonella* gallinarum [31], Salenvac vaccine [32], Typhoid vaccine Vi polysaccharide, Avirpo *Salmonella* Vac E, and so on. However, the scarcity of licensed vaccines targeting specific disease treatments underscores the pressing need for novel solutions. Given the substantial demand for treating poultry disease infections and the longstanding industrial demand for *Salmonella* vaccines, BG vaccines emerge as promising candidates. With their potential to offer both safety and efficacy, BG vaccines hold significant development prospects in addressing poultry diseases and meeting industrial demands.

**Scheme 1:**
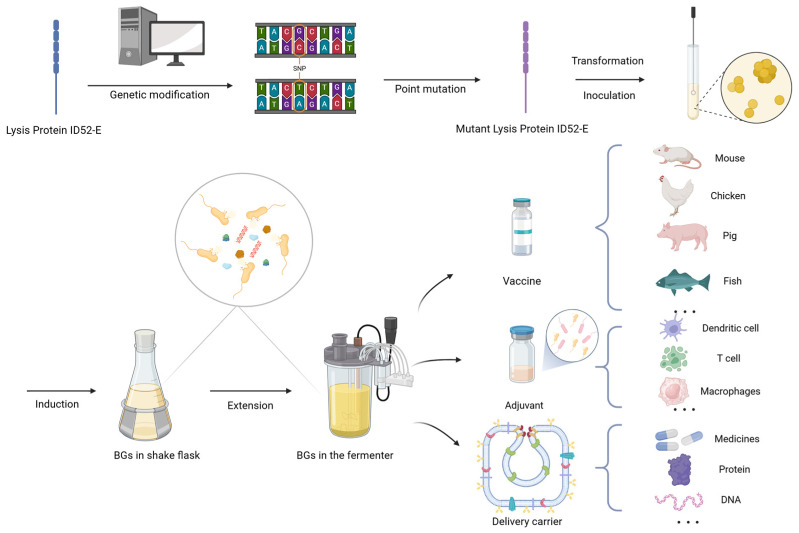
Schematic diagram of modifying lysis protein ID52-E to efficiently prepare BGs for subsequent applications.

In our study, on the basis of previous studies of the lysis effect of ID52-E, which was relatively more effective than that of φX174-E, several protein sequence modifications were made to optimize lysis function. The site-directed mutation based on the phage ID52 lysis protein E in *E. coli* resulted in the generation of 13 mutants, among which a mutant exhibiting the most potent lysis effect was identified. Subsequently, to enhance the lysis rate, the culture medium and inducer concentration were optimized. Additionally, efforts were made to augment the yield of BGs by culturing bacteria in a fermentation tank and optimizing the conditions. This approach was applied to prepare EcN ghosts, SP ghosts, and SC ghosts, leading to further enhancements in the yield and productivity of EcN ghosts (Scheme 1). Finally, to investigate whether the mechanism of action of the gene ID52-E resembled that of the gene φX174-E, the molecular chaperone genes *slyD* and *dnaJ* were knocked out, and integral membrane enzyme gene *mraY* was overexpressed. However, the improvement in yield and production of BGs provides a solid foundation for industrial production of BGs and promises to be a natural adjuvant for future vaccine formulations and cancer treatments.

## 2. Materials and Methods

### 2.1. Bacterial Strains, Plasmids, and Growth Conditions

The bacterial strains and plasmids utilized in this study were summarized in Table S1. All bacteria cultures were incubated at 37 °C. Bacteria were routinely cultured in either Luria-Bertani (LB) broth or 2xYT (Yeast Extract Tryptone) media.

### 2.2. Evolutionary Tree Analysis

To discern the distinctions between φX174-E and ID52-E, an evolutionary tree analysis was conducted to elucidate the relationships between the two lysis proteins. Initially, we collated the amino acid sequences of *E. coli* phage lysis E, utilizing specific identifiers available from The National Center for Biotechnology Information (NCBI). This process involved meticulous organization of the amino acid sequences alongside their respective species names and identification (ID) names, which were curated utilizing a text editor (http://gitee.com/cxasm/notepad--, accessed on 10 April 2024). Subsequently, the compiled amino acid sequences were subjected to multiple sequence alignment using the Molecular Evolutionary Genetics Analysis (MEGA) computer program (version 11.0.1; MEGA software, Cypress, TX, USA). The statistical robustness of the resultant evolutionary tree was evaluated employing 1000 bootstrap replicates. Finally, the developmental tree was refined for visual clarity and aesthetic appeal utilizing Evolview (http://www.evolgenius.info/evolview-v2/#login, accessed on 10 April 2024).

### 2.3. Construction of Plasmids

The cloning reactions pertaining to single-point mutations were executed using the Restriction-Free (RF) method [33]. Various lysis protein mutants were generated via site-directed mutagenesis employing RF cloning, utilizing the araC-ParaBAD-ID52-E plasmid as the template. Enzymes and reagents necessary for Restriction-Free (RF) were obtained from Thermo Fisher Scientific (Waltham, MA, USA). The fragment encompassing the lysis gene φX174-E was obtained by polymerase chain reaction (PCR) amplification utilizing the φX174-E-F/φX174-E-R primers, with plasmid pBV220-φX174-E as the template. Subsequently, the Seamless Cloning Kit (Sangon Biotech, Shanghai, China) was utilized to insert the φX174-E fragment into the araC-ParaBAD-ID52-E vector backbone. The resultant plasmid was designated as araC-ParaBAD-φX174-E. Furthermore, the *mraY* gene fragment was amplified from the *Bacillus subtilis* genome, and the pLysS-*mraY* expression vector was constructed accordingly. The aforementioned plasmids were subsequently transformed into *E. coli* DH5α competent cells (TsingKe, Beijing, China), followed by the selection of single clones post-transformation. A comprehensive list of all primers employed in these cloning procedures is listed in Table S2.

### 2.4. Lysis Curve Assay

The araC-ParaBAD-ID52-E mutant plasmids were transfected into EcN *∆araBAD::FRT*. Subsequently, the bacteria were cultured until reaching the same OD_600_ value, following which the expression of lysis proteins was induced by the addition of 0.25 mg/mL of L-arabinose. The effect of bacterial lysis was assessed by monitoring the OD_600_ at hourly intervals and constructing lysis curves. To identify the most efficacious protein resulting from the current mutation, curves were meticulously compared, facilitating the screening of the optimal protein.

### 2.5. Fermenter Assay

The experiments involving inducer and medium optimization were initially conducted in conical flasks and subsequently translated to the fermenter setup. L-arabinose served as the inducer, with concentration gradients of 0.25 mg/mL, 0.5 mg/mL, 1 mg/mL, and 1.5 mg/mL employed for optimization. Four liquid media, LB, 2xYT, Terrific Broth (TB), and Super Broth (SB), were chosen for evaluation. The bacteria were cultured to the same OD_600_ and then induced to screen out the most favorable conditions for BG production.

In order to obtain the optimal conditions for the BGs in the fermenter, a strategy involving pH and DO adjustment was implemented. Fermentation was conducted at a temperature of 37 °C, with a stirring speed of 500 rpm. The pH was modulated across three gradients of 6.0, 6.5, and 7.0, while DO was adjusted to three levels: 35%, 40%, and 45%, thus facilitating single-variable control. DO regulation was achieved by incrementally adjusting the stirring speed, with a cap set at 800 rpm to prevent excessive agitation.

Following the screening process to determine the optimal fermentation conditions, a comparative analysis was performed between the conventional lysis protein φX174-E and the screened optimal lysis protein. Cultures of EcN *∆araBAD::FRT* harboring the aforementioned plasmids were cultured overnight, and the seed solution was inoculated at 5% (*v*/*v*) into the fermenter with varying OD_600_ for induction. The highest starting induction OD_600_ capable of yielding BGs was systematically explored to refine the induction process.

### 2.6. Characterization of the Yield of BGs

To quantify the yield of BGs, ghost samples were concurrently collected and processed. Following collection, the samples underwent thorough washing and subsequent staining with propidium iodide (PI). Subsequently, the stained samples were fixed and subjected to confocal imaging utilizing a confocal microscope (TCS SP8, Leica, Wetzlar, Germany). In order to accurately discern the quantitative data of BG production at various time points, flow cytometric parameters were employed. Given that the induced bacterial cultures contain a mixture of live bacteria, those yet to form BGs, and dead bacteria, the anionic fluorescent dye bis-(1,3-dibu-tylbarbituric acid) trimethine oxonol [DiBAC4 _(3)_] (Yeasen Biotechnology, Shanghai, China) was utilized for direct BGs detection [34]. Samples were collected at distinct time points during BG generation, and subsequent to labeling, the fluorescence intensity was quantified using a NovoCyte flow cytometer (ACEA NovoCyte D2060R, Agilent Biosciences, Santa Clara, CA, USA). This comprehensive approach facilitated the precise assessment of BG production dynamics over time.

### 2.7. Scanning Electron Microscopy (SEM) and Transmission Electron Microscopy (TEM)

BGs were subjected to electron microscopy for detailed structural analysis. Initially, the BGs were washed and fixed using a 2.5% glutaraldehyde fixative solution overnight. Following fixation, the BGs underwent another round of washing and were subsequently resuspended in deionized water. The bacterial suspension was then carefully applied onto a cell strip, enveloped in filter paper, and arranged in order. Dehydration was achieved by sequentially immersing the specimen in 70%, 85%, and 95% ethanol for 15 min each, followed by a final 15 min immersion in 100% ethanol. The samples were then critically dried using a critical point dryer (Autosamdri-815, Series A, Tousimis, Rockville, MD, USA), gold-coated, and examined using SEM (Hitachi SU8010, Chiyoda, Japan).

Similarly, transmission electron microscopy (TEM) was employed for structural examination prior to BG treatment and fixation overnight. The procedure commenced by placing a carbon-coated copper mesh onto a dry and clean filter paper, followed by the careful application of bacterial solution onto the front side of the carbon-coated copper mesh using a pipette. The mesh was then left at room temperature for 10 min to allow for bacterial adherence. Excess bacterial solution was removed by blotting with paper towels, and the mesh was gently air-dried. Subsequently, the front side of the copper mesh was coated with a 3% phosphotungstic acid staining solution and left at room temperature for 5 min to stain the bacteria. Excess dye was absorbed using blotting paper. Finally, the samples were observed under a transmission electron microscope (FEI Tecnai G2F30, Hillsboro, OR, USA)

### 2.8. Construction of SP and SC BGs

To investigate the applicability of the lysis proteins to *Salmonella*, two kinds of *Salmonella* were utilized, *Salmonella enterica* subsp. *enterica* serovar ppullorum (SP) and *Salmonella enterica* subsp. *enterica* serovar choleraesuis (SC). The araC-ParaBAD-ID52-E-W4A plasmids, identified through screening, were introduced into the aforementioned pathogenic bacteria. Subsequently, the bacteria were cultured until they reached a specified optical density at OD_600_ for induction, and observations were made to ascertain the production of BGs. Lysis curves for both bacterial strains were constructed based on OD_600_ measurements, while the morphological characteristics of the bacteria were examined and observed using SEM and TEM. This comprehensive approach facilitated the assessment of the efficacy and suitability of the lysis proteins for *Salmonella* strains SP and SC, encompassing both functional and morphological analyses.

### 2.9. Knockout slyD and dnaJ and Overexpresses mraY

CRISPR/Cas9 gene targeting was utilized to engineer EcN-engineered bacteria deficient in *slyD* and *dnaJ* genes, respectively. Initially, Cas9 was transferred into EcN, and recombinant-positive clones were identified via kanamycin. Subsequently, the DNA sequences corresponding to *slyD* (WP_000861334.1) and *dnaJ* (WP_001118465.1) were located within the EcN genome (GenBank: NZ_CP007799.1). Gene-specific single guide RNAs (sgRNA) of two target genes were designed using the software (http://crispor.tefor.net/). The sgRNA primers for the two target genes were synthesized according to the designed method named SN20-F/SN20-R and DN20-F/DN20-R, respectively (upper case N20 sequence of 20 bp). Homology arms of 1000 bp upstream and downstream of the target gene were selected for the construction of the target plasmid, and the designed amplification primers were *slyD*-up1000-F/*slyD*-up1000-R, *slyD*-dn1000-F/*slyD*-dn1000-R, *dnaJ*-up1000-F/*dnaJ*-up1000-R, *dnaJ*-dn1000-F/*dnaJ*-dn1000-R, and the primers are shown in Table S2.

The N20 sequence was first constructed via RF cloning, followed by the construction of the sgRNA recombinant plasmid through seamless cloning of the upstream and downstream 1000 bp regions. Subsequently, the sgRNA recombinant plasmid was introduced into the EcN-competent cell containing Cas9 for gene knockdown. Positive clones were verified using ∆*slyD*-F/∆*slyD*-R, ∆*dnaJ*-F/∆*dnaJ*-R. Finally, the engineered EcN strains with *slyD* and *dnaJ* deletion were designated as EcN ∆*slyD* and EcN ∆*dnaJ*, respectively. Plasmids encoding lysis proteins were transfected into the aforementioned engineered bacteria, and the lysis curves were generated to assess their efficacy.

Furthermore, the plasmids pLysS-mraY and araC-ParaBAD-ID52-E were transferred into EcN *∆araBAD::FRT*. Lysis curves were determined for EcN *∆araBAD::FRT* containing the two plasmids described above.

### 2.10. Statistical Analysis

All statistical analyses were performed with GraphPad Prism 9.0.0 software. Analysis of variance and Tukey–Kramer test were used to compare means among three or more groups. The data are presented as mean ± SD. All experiments were repeated at least three times.

## 3. Results

### 3.1. Evolutionary Tree Analysis of Phage Lysis Protein E

A phylogenetic tree of lysis proteins was constructed to elucidate the evolutionary diversity within this protein family. Analysis at the amino acid level, as depicted in Figure S1, highlights that the two proteins ID52-E and φX174-E exhibit a relatively distant relationship. However, through phylogenetic analysis, it becomes evident that the genetic affinity of ID52-E is closer to G4-E, whereas φX174-E shows a closer genetic relationship to α3-E than to ID52-E. Notably, all four proteins are presently available in the laboratory. In comparative experiments involving the originally studied ID52-E and φX174-E, it is important to consider their distinct genetic relationships and potential differences in mechanisms (Figure S1). Despite both proteins being capable of inducing cleavage, their effects may vary, and they may target different sites or exhibit different binding affinities. Therefore, their comparative analysis may shed light on their unique functionalities and mechanisms of action.

### 3.2. Construction of Mutant Plasmids and Lysis Curve Assay

Upon the foundation of araC-ParaBAD-ID52-E, various mutations were introduced into ID52E, resulting in the generation of 13 mutants, as delineated in Figure S2. The positive clones were selected based on growth on LB plates and subsequently validated through PCR analysis (Figure S3A).

**Figure 1:**
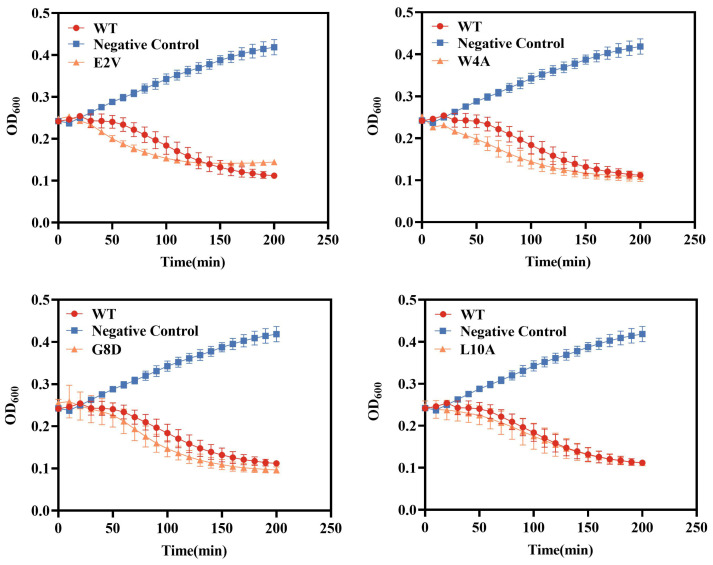
The lysis curves of mutant active EcN. The growth curves of at least five replicates of each mutant were monitored using OD_600_.

The efficiency of the phage lysis protein E was evaluated through optical density measurements at OD_600_ nm. Induction followed by lysis was monitored by observing the decrease in OD_600_ over time, with the onset of lysis defined as the time point at which OD_600_ began to decline. To facilitate a comparative analysis of the lysis protein mutants, induction was standardized to the same OD_600_ value. The most promising results from the alanine scanning were integrated into the amino acid sequence of φX174-E to ID52-E. Certain mutations were introduced to align with amino acid disparities between the two proteins. As observed in previous reports, the lysis proteins initially exhibited a transient rise within the first 20 min, followed by a subsequent decline [35]. Notably, most site mutations retained lysis activity, except for mutations at positions 7 (S7W), 61 (C61S and C61S&C93S), 66 (F66C and F66C&S83C), and the previously identified mutation at position 21 (P21A) [36], which led to complete loss of activity (Figure S4). These outcomes are likely attributed to specific site characteristics and the size or properties of the mutated amino acids. Conversely, mutations such as R3A, I9T, R33K, L37S, S83C, and C93S exhibited minimal impact on lysis activity (Figure S5), suggesting that these sites may not be critical. Notably, four mutant lysis proteins (E2V, W4A, G8D, L10A) markedly accelerated the onset of lysis. To ensure experimental accuracy, each experiment was repeated five times, with W4A emerging as the mutant with the fastest lysis rate at the same time point (Figure 1).

### 3.3. Fermenter Assay

**Figure 2:**
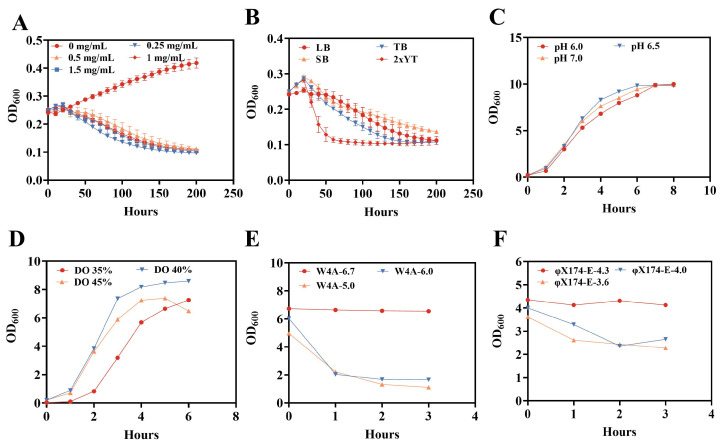
Optimization and lysis curve of BGs in fermentation tanks. The lysis curves of lysis proteins ID52-E-W4A and φX174-E in fermentation tanks. (**A**) The concentrations of L-arabinose were 0 mg/mL, 0.25 mg/mL, 0.5 mg/mL, 1 mg/mL, and 1.5 mg/mL, respectively. (**B**) The culture media were LB, TB, 2xYT, and SB, respectively. (**C**) The pH was 6.0, 6.5, and 7.0, respectively. (**D**) The DO was 35%, 40%, and 45%, respectively. (**E**) The initial induction OD_600_ values were 6.7, 6.0, and 5.0. (**F**) The initial induction OD_600_ values were 4.3, 4.0 and 3.6.

Within the lysis plasmids araC-ParaBAD-φX174-E and araC-ParaBAD-ID52-E-W4A, the expression of the lysis protein was regulated by an arabinose-inducible promoter. Notably, different concentrations of L-arabinose exhibited no significant effect on the lysis efficiency of ID52-E-W4A, as demonstrated by measuring the EcN lysis curve at identical initial induction OD_600_ (Figure 2A). To reduce costs, a final concentration of 0.25 mg/mL L-arabinose was deemed optimal for subsequent experiments.

While LB medium is commonly used in laboratory settings, its nutritional content may not be entirely conducive to BG preparation. Therefore, alternative culture media were considered to ensure optimal growth conditions for BG production. Among these, 2xYT medium emerged as highly suitable for BG preparation, exhibiting a faster BG production rate compared to other media under identical initial induction OD_600_ and time conditions (Figure 2B). Consequently, 2xYT medium was selected for subsequent fermenter experiments.

During fermentation, pH and dissolved oxygen (DO) levels fluctuate and must be carefully controlled to ensure optimal bacterial growth. Through optimization experiments, it was determined that bacteria exhibited optimal growth at pH 6.5 and DO 40%. Accordingly, pH and DO levels were maintained at these values throughout fermenter experiments (Figure 2C,D).

While bacteria can grow to an OD_600_ of 10 under the various conditions mentioned above, BG production necessitates induction during the logarithmic growth phase. To ascertain the highest initial induction OD_600_ for BG production in the fermenter, lysis curves were measured at different initial induction OD_600_ values. Comparative analysis between EcN strains containing araC-ParaBAD-φX174-E and araC-ParaBAD-ID52-E-W4A lysis plasmids revealed that under the influence of ID52-E-W4A protein, the highest initial induction OD_600_ can reach 6.0, with a final lysis OD_600_ of approximately 1.6 (Figure 2E). In contrast, the highest initial induced OD_600_ of φX174-E lysis protein is only 4.0, resulting in a final lysis OD_600_ of about 2.6 (Figure 2F). These findings indicate that the yield of BGs generated by ID52-E-W4A substantially surpasses that of φX174-E.

### 3.4. The Yield of EcN BGs by Fluorescence Microscopy and Flow Cytometry

**Figure 3:**
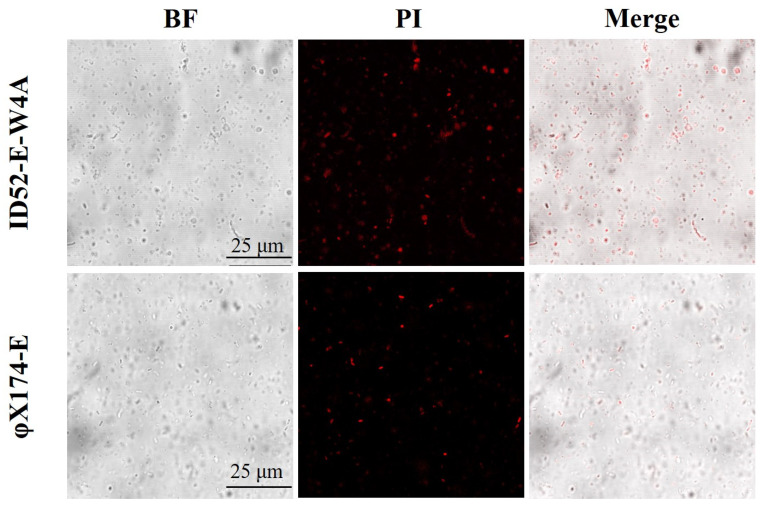
Fluorescence microscopy to determine the yield of BGs. The red fluorescence (PI) efficiency of BGs staining mediated by ID52-E-W4A was essentially higher than that of BGs mediated by φX174-E.

**Figure 4:**
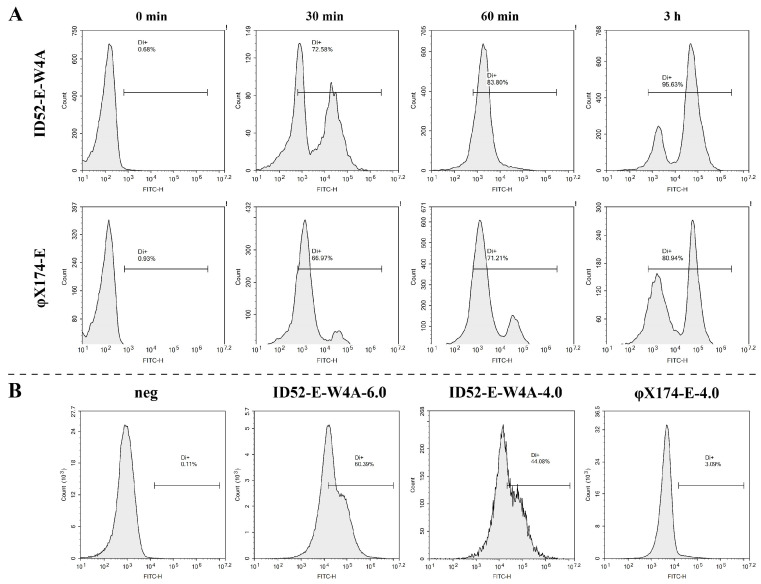
Flow cytometry to determine the yield of BG in shake flasks and fermenters. (**A**) Flow cytometry was used to detect the effect of BGs at different times on DIBAC_4 (3)_ fluorescence. The control group did not use DIBAC_4 (3)_. (**B**) Flow cytometry was used to detect the effect of two lysis proteins on DIBAC_4 (3)_ fluorescence induced by OD_600_ BGs at different starting times. The control group did not use DIBAC_4 (3)_.

Upon initial examination of propidium iodide (PI) staining using a confocal microscope, it was observed that the red fluorescence intensity associated with BGs mediated by ID52-E-W4A was significantly greater compared to BGs mediated by φX174-E (Figure 3). To assess the formation rate of BGs, DIBAC4_(3)_ dye was utilized, which selectively binds to bacteria with disrupted membranes. As illustrated in Figure 4, the comparison was conducted at identical time points. EcN *∆araBAD::FRT* harboring the araC-ParaBAD-φX174-E and araC-ParaBAD-ID52-E-W4A plasmids were evaluated for BGs formation. Bacteria that did not undergo BG induction exhibited minimal DI+ fluorescence signals, indicative of intact and undamaged cell membranes, with only 0.7% and 1.0% fluorescence observed. Over time, the rate of ghost formation mediated by both lysis proteins, ID52-E-W4A and φX174-E, increased steadily. After 3 h of lysis in a shake flask, the formation rates of BGs induced by ID52-E-W4A and φX174-E reached 95.6% and 81.0%, respectively (Figure 4A). Notably, in the fermenter experiments, the yield of ID52-E-W4A-induced BGs reached 67.0%, while the yield of φX174-E induced BGs was markedly lower at 3.1% (Figure 4B). These findings underscore the superior efficiency of ID52-E-W4A in facilitating BG formation. Moreover, the productivity of ghost formation mediated by ID52-E-W4A exceeded that of φX174-E at each time point, further highlighting its enhanced efficacy.

### 3.5. Characterization of EcN BGs by SEM and TEM

**Figure 5:**
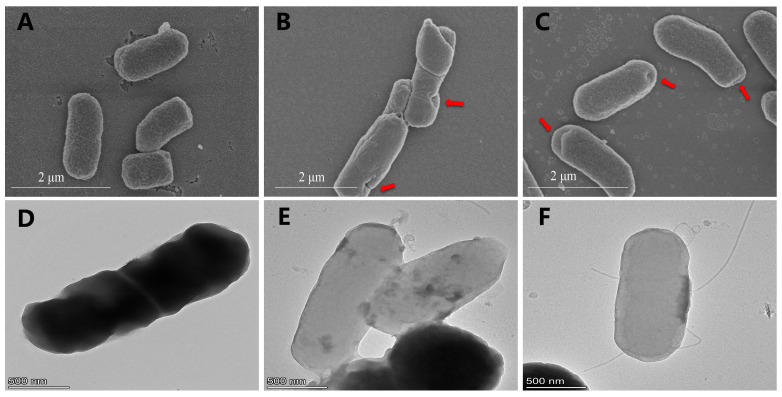
Morphological observation of EcN BGs by SEM and TEM. SEM of (**A**) intact morphology of wild-type EcN, and (**B**,**C**) EcN ghosts formed after lysis induced by plasmid araC-ParaBAD-ID52-E-W4A. TEM of (**D**) intact morphology of wild-type EcN, and (**E**,**F**) EcN ghosts formed after lysis induced by plasmid araC-ParaBAD-ID52-E-W4A. The red arrow indicates the transmembrane tunnels.

Scanning electron microscopy (SEM) was employed to examine the morphological features of bacteria, revealing that the surface morphology of BGs closely resembles that of intact bacteria (Figure 5A). Notably, the cell morphology of bacterial cells remains largely preserved. However, regions of the cell membrane where lysis pores are generated exhibit inward dents, indicating their penetration into the cell cavity (Figure 5B,C). These pores result from osmotic pressure differentials between the interior and exterior of the cell. As the cell contents are released, the cell may eventually collapse entirely. The cells depicted here are in a state prior to complete collapse. Consistent with previous literature, lysis pores typically manifest at the equator or poles of the cell [37].

To corroborate the shedding of bacterial contents, transmission electron microscopy (TEM) was conducted. Untreated EcN cells appear dark black after staining, indicative of the presence of bacterial contents within the cell cavity (Figure 5D). Conversely, the cell cavity where BGs formed following induced lysis by protein E exhibits a lighter coloration, reflecting the absence of cytoplasm and DNA within the interior. The loss of cytoplasm observed in TEM appears relatively extensive, yet the overall bacterial morphology remains largely unchanged (Figure 5E,F).

### 3.6. Expanded Application of ID52-E-W4A on Production of SP and SC BGs

**Figure 6:**
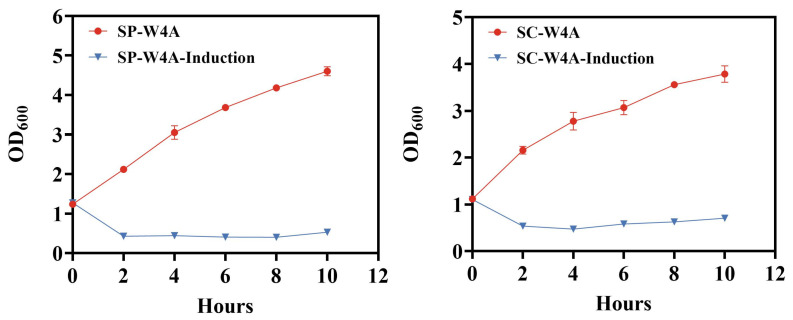
The lysis curves of SP and SC containing plasmid araC-ParaBAD-ID52-E-W4A.

**Figure 7:**
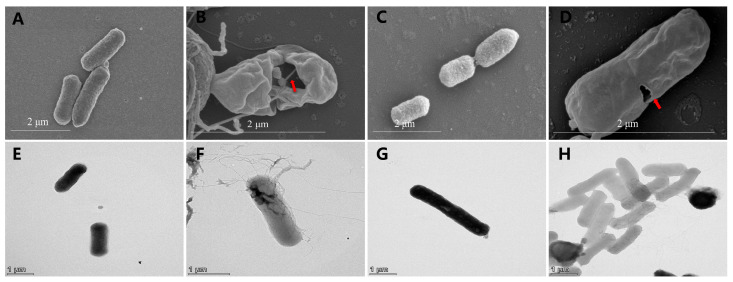
Morphological observation of SP BGs and SC BGs induced by plasmid araC-ParaBAD-ID52-E-W4A using SEM and TEM. SEM of (**A**) intact morphology of wild-type SP, (**B**) SP ghosts formed after lysis, (**C**) intact morphology of wild-type SC, and (**D**) SP ghosts formed after lysis. TEM of (**E**) intact morphology of wild-type SP, (**F**) SP ghosts formed after lysis, (**G**) intact morphology of wild-type SC, and (**H**) SP ghosts formed after lysis. The red arrow highlights the presence of transmembrane tunnels within the structures.

To ascertain the applicability of the ID52-E-W4A protein recombinantly expressed under the control of araC-ParaBAD in the generation of *Salmonella* in addition to *E. coli* in the preparation of BGs, we extended our investigation to include SP and SC. Upon induction with the same initial OD_600_, both SP and SC harboring the ID52-E-W4A lysis protein underwent cleavage, resulting in a reduction in OD_600_ to approximately 0.5 (Figure 6). Scanning electron microscopy revealed that both SP BGs and SC BGs retained intact cell morphology and layered structures, as compared to untreated SP and SC counterparts. Notably, transmembrane tunnels induced by the lysis protein were observed at either the equator or pole of the bacterial cell (Figure 7A–D). Furthermore, transmission electron microscopy analysis demonstrated the displacement of cellular contents within SP BGs and SC BGs (Figure 7E–H). These findings collectively affirm the feasibility of utilizing the ID52-E-W4A protein in the preparation of *Salmonella* BGs.

### 3.7. Exploration of the Mechanism of ID52-E

**Figure 8:**
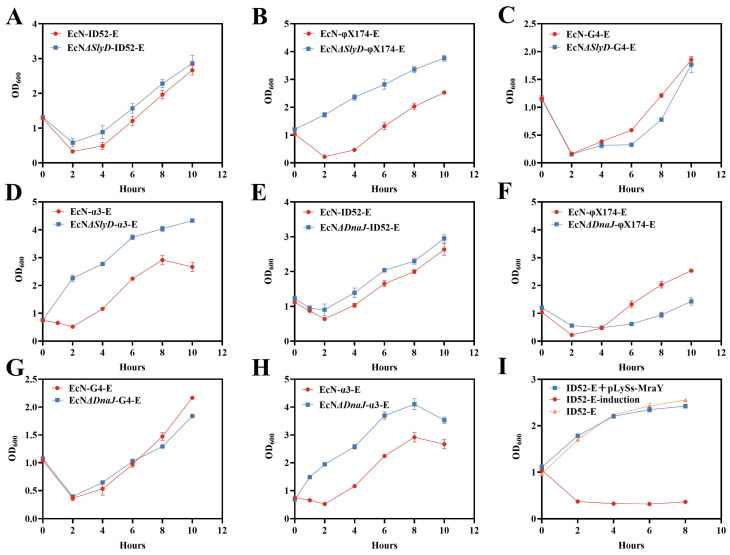
The lysis curves of EcN ∆*slyD*, EcN ∆*dnaJ*, and overexpressed *mraY*. (**A**–**D**) EcN ∆*slyD* containing lysis gene ID52-E, φX174-E, G4-E, and α3-E, respectively. (**E**–**H**) EcN ∆*dnaJ* containing lysis gene ID52-E, φX174-E, G4-E, and α3-E, respectively. (**I**) The lysis curves of overexpressed *mraY* and lysis protein ID52-E.

The gene *slyD* (sensitive to lysis D) was initially identified as the first but not the last cellular target of φX174-E, although subsequent investigations have revealed additional targets. MS2-L, a member of the prototoxin family within the bacteriophage single-stranded RNA group, shares similarities with φX174-E. Notably, the molecular chaperones essential for φX174-E and MS2-L-mediated lysis are encoded by *slyD* and *dnaJ* genes, respectively. To ascertain whether the molecular chaperone of ID52-E is analogous to *slyD* or *dnaJ*, we employed CRISPR/Cas9 gene targeting to disrupt both genes in EcN. Plasmids encoding the lysis protein were then introduced into EcN strains lacking either *slyD* (EcN ∆*slyD*) or *dnaJ* (EcN ∆*dnaJ*). The progression of bacterial lysis was monitored through lysis curve measurements (Figure 8A–H). As depicted in Figure 8A–E, the results indicated that bacterial lysis persisted even after the knockout of *slyD* or *dnaJ*, suggesting that neither gene is necessary for ID52-E to exert its lysis function.

Previous studies have proven that protein E serves as a scaffold of *mraY*, a conserved membrane-embedded enzyme crucial for catalyzing peptide scaffold synthesis within the cell wall biosynthesis pathway [38,39]. To validate whether *mraY* serves as a cellular target for ID52-E and recognize the impracticality of knocking out *mraY* in bacteria, we opted to overexpress *mraY* in bacterial cells and observe its impact on lysis activity. The introduction of the pLysS-*mraY* plasmid resulted in a distinct band at 972 base pairs (Figure S3B). Subsequent experimentation revealed that upon induction of plasmid function and overexpression of *mraY*, bacterial lysis activity diminished (Figure 8I), suggesting that *mraY* likely serves as a cellular target for ID52-E.

## 4. Discussion

Vaccination stands as a widely employed method in daily life for preventing various infectious diseases. As a potential vaccine, BG vaccines find application not only in humans [4] but also in the preparation of pathogenic bacteria ghosts, particularly in poultry ghost vaccines, including bovine pathogenic *Escherichia coli* ghost [40], *Vibrio cholerae* ghost [41], bovine *Pasteurella multocida* ghost [42], *Edwardsiella tarda* ghost [43], *Aeromonas hydrophila* ghost [44], and *Vibrio alginolyticus* ghost [45], among others. Furthermore, BGs have increasingly been utilized as carriers for nucleic acids, proteins, and chemical drugs. Notably, EcN ghosts have been developed and utilized as delivery vehicles or adjuvants. For instance, EcN ghosts releasing ciprofloxacin can effectively eliminate bacteria within macrophages through targeted delivery [7]. Additionally, as adjuvants, EcN ghosts can synergize with oxaliplatin to enhance the immunogenicity of the anti-cancer response.

In recent years, the challenge of enhancing BG production has posed a significant concern among scholars, with BGs yet to receive official approval and commercialization [23]. There is a growing demand for high-efficiency BGs with increased yield and productivity, and as of now, no large-scale fermentation experiments have been conducted with EcN BGs. It is anticipated that by delving into the superior lysis effect of ID52-E over φX174-E, advancements can be made in this area. In our study, a mutant (ID52-E-W4A) exhibiting enhanced lysis effects compared to ID52-E was identified through point mutations. The substitution of tryptophan with alanine at position 4 altered the size of the side chain, potentially facilitating membrane insertion. However, the screening of efficient mutants is constrained by time and methods. Consequently, there is a need to utilize bioinformatics to simulate the formation of different aggregates and assess the stability among aggregates for carrying out diverse mutations.

Scientists have traditionally utilized serial dilution and plate counting methods to assess bacterial lysis activity before and after treatment [23]. The inactivation efficiency assay of different lysis proteins was determined (Table S4). However, these methods do not directly measure the yield of BGs but rather the lethality rate of bacteria. In our study, we employed flow cytometry to obtain specific BG measurements, allowing for a more accurate assessment of BG yield. Initially, we conducted experiments in a smaller fermentation tank to explore the feasibility of efficiently preparing BGs. The initial OD_600_ mediated by the gene ID52-E-W4A was approximately 6.0, while the final OD_600_ was around 1.6. The results from the fermentation tank experiment revealed that the lysis effect of ID52-E-W4A surpassed that of φX174-E, evident in both the initial induction OD_600_ and the OD_600_ achieved after the final lysis, as well as in the productivity of the fermentation tank. The yield of EcN ghosts mediated by ID52-E-W4A reached a maximum of 0.223 g/L. However, it is essential to consider the nutrient composition of the culture medium to support large-scale bacterial growth and induce the logarithmic growth phase of bacteria effectively. Further investigation is warranted to determine whether other media suitable for high-density fermentation can provide continuous and rapid energy for bacterial growth while inducing lysis proteins at high OD_600_ levels to efficiently prepare BGs.

Studies have indicated that the cell lysis mechanism mediated by φX174-E protein is associated with cell division, and its kinetics are species-specific, likely similar to that of the ID52-E protein [21,23,46]. The maximum initial lysis OD_600_ and minimum lysis OD_600_ vary significantly among different host microbes and need to be optimized according to the specific bacterial strains [23]. In addition to Escherichia coli ghosts, Salmonella enteritidis ghosts, and Salmonella typhimurium ghosts were also prepared using ID52-E-W4A in our previous study. Further investigation into the preparation of high-efficiency Salmonella BGs and expanded research on their immune effects are warranted. The expectation for the use of the BG vaccine can be by the mucosal route or by injection or oral administration.

Throughout the duration of the experiment, our focus centered on elucidating the mechanism of action underlying the gene ID52-E. Central to our inquiry was the conjecture regarding its potential association with the target and molecular chaperone of φX174-E. Existing research posited the involvement of the transmembrane proline residue P21 in E-mediated protein lysis, suggesting that the cis-trans isomerization of this residue could constitute a pivotal step in E-mediated lysis [36]. Additionally, the indispensability of the chaperone SlyD (sensitivity to lysis D) in maintaining the stability of φX174-E was underscored, ostensibly by shielding the toxin from proteolytic degradation. Activation of φX174-E was contingent upon interaction with the bacterial chaperone SlyD, which concurrently hindered the enzymatic activity of cell wall precursor-forming enzymes [47]. AnnA K et al. [48] further elucidated the role of protein E in facilitating the formation of a transmembrane YES complex comprising mra**Y**, protein **E**, and **S**lyD. Notably, protein E was found to impede peptidoglycan biosynthesis by obstructing the active site of mraY, thereby impeding lipid I production. The L protein of single-stranded RNA bacteriophage MS2 was demonstrated to induce lysis of *E. coli*, a process reliant on the host molecular chaperone *dnaJ* [49]. Subsequent verification experiments revealed the loss of lysis activity following the P21A mutation in ID52-E, yet lysis persisted upon knockout of *slyD* and *dnaJ*, suggesting that ID52-E may operate independently of these chaperones. However, the necessity of partner assistance for its activation or its potential for autonomous activation warrants further exploration. Furthermore, overexpression of *mraY* led to a loss of bacterial activity, indicative of ID52-E potentially inhibiting *mraY*, thereby implicating *mraY* as its likely target.

## 5. Conclusions

In our investigation, we identified protein ID52-E-W4A as exhibiting the most pronounced lysis effect among 13 mutants derived from the phage ID52 lysis protein E. Subsequently, this protein variant was utilized in the preparation of EcN and *Salmonella* BGs. Our study represents a pioneering effort to establish a highly efficient method for the recombinant expression of BGs under the regulatory control of araC-ParaBAD within a fermentation tank. This advancement significantly enhances both the yield and productivity of BGs in EcN, thereby expanding the potential applications of *Salmonella* BGs and laying a solid foundation for their large-scale production and diverse utilization. Moreover, our findings indicate that ID52-E operates independently of the chaperones *slyD* and *dnaJ*, suggesting that *mraY* likely serves as the target of ID52-E. These insights provide valuable directions for further elucidating the mechanism of action underlying the lysis protein ID52-E.

## Data Availability

Data are contained within the article.

## Supplementary Materials

The following supporting information can be downloaded at: https://www.mdpi.com/article/10.3390/vaccines12050472/s1, Table S1: Materials including strains and plasmids used in our study; Table S2: Materials including primers used in our study; Table S3: Agricultural productivity and yield of two lysis proteins; Figure S1: The evolutionary tree analysis of Enterobacteriaceae phage lysis gene E; Figure S2: Comparison of the amino acid sequences of wild-type and mutant of phage ID52 lysis protein E; Figure S3: Colony analysis by agarose gel electrophoresis; Figure S4: The lysis curves of EcN mutated to lose activity; Figure S5: The lysis curves of the EcN mutant was not significantly affected; Table S4: The inactivated efficiency of different lysis protein E in EcN; Figure S6: The expression of lysis protein ID52-E-W4A was verified by western blot.
