# Supplementary material for: Construction and Mechanism Exploration of Highly Efficient System for Bacterial Ghosts Preparation Based on Engineered Phage ID52 Lysis Protein E
Source: Vaccines (Basel). 2024 Apr 28;12(5):472. doi: 10.3390/vaccines12050472 (PMC11126076; doi:10.3390/vaccines12050472)
Supplement: Supplementary file 1 [file vaccines-12-00472-s001.zip › vaccines-2926983-supplementary.pdf]

Table S1. Materials, including strains and plasmids used in our study.

Strains	Description	Source
DH5α	Host cells for plasmid amplification	TsingKe, Beijing, China
EcN *ΔaraBAD::FRT*	Deletion of *araBAD* gene and insertion of FRT locusin *E. coli* Nissle 1917	Our lab
SP, *Salmonella* pullorum	*Salmonella enterica subsp. enterica serovar* pullorum ATCC9120	Our lab
SC, *Salmonella* choleraesuis	*Salmonella enterica subsp. Enterica serovar* choleraesuis ATCC13312	Our lab
EcN *ΔslyD*	Deletion of *slyD* gene in EcN	This study
EcN *ΔdnaJ*	Deletion of *dnaJ* gene in EcN	This study
**Plasmids**		
araC-ParaBAD-ID52-E	The template for constructing gene vectors for mutant genes	Our lab
pBV220-φX174-E	The template for constructing expression vector of gene φX174-E	Our lab
araC-ParaBAD-ID52-E-W4A	Lysis plasmid used in this study	This study
araC-ParaBAD-ID52-E-C61S	The mutant gene of ID52-E	Our lab
araC-ParaBAD-ID52-E-C93S	The mutant gene of ID52-E	Our lab
araC-ParaBAD-ID52-E-C61S&C93S	The mutant gene of ID52-E	Our lab
araC-ParaBAD-φX174-E	Lysis plasmid used in this study	This study
pLysS-pHLIP	The template for constructing expression vector of *mraY*	Our lab
pCas	Plasmid for CRISPR-Cas9	Our lab
pTargetF	Plasmid for CRISPR-Cas9	Our lab

Table S2. Materials, including primers used in our study.

Primers	
φX174-E-F	TCGAGCTCTAAGGAGGTTATAAAAAATGGTACGCTGGACTTTGTG
φX174-E-R	TCAAACTGCGGATGAGACCACTCCTTCCGCACGTAATTT
ID52E-F	ATGGAACGCTGGACCTTAAGC
ID52E-R	ACCGCCAGTACCGCGAC
E2V-F	GGTTATAAAAAATGGTACGCTGGACCTTAAGCGGCATT
E2V-R	AATGCCGCTTAAGGTCCAGCGTACCATTTTTTATAACC
R3A-F	TATAAAAAATGGAAGCGTGGACCTTAAGCGGCATTCTG
R3A-R	CAGAATGCCGCTTAAGGTCCACGCTTCCATTTTTTATA
W4A-F	AAAAAATGGAACGCGCGACCTTAAGCGGCATTCTGGCG
W4A-R	CGCCAGAATGCCGCTTAAGGTCGCGCGTTCCATTTTTT
S7W-F	ATGGAACGCTGGACCTTATGGGGCATTCTGGCGTTTCT
S7W-R	AGAAACGCCAGAATGCCCCATAAGGTCCAGCGTTCCAT
G8D-F	GAACGCTGGACCTTAAGCGATATTCTGGCGTTTCTGCT
G8D-R	AGCAGAAACGCCAGAATATCGCTTAAGGTCCAGCGTTC
I9T-F	CGCTGGACCTTAAGCGGCACCCTGGCGTTTCTGCTTCT
I9T-R	AGAAGCAGAAACGCCAGGGTGCCGCTTAAGGTCCAGCG
L10A-F	CCTTAAGCGGCATTGCGGCGTTTCTGCTTCTGCTGAGC
L10A-R	GCTCAGCAGAAGCAGAAACGCCGCAATGCCGCTTAAGG
P21A-F	CTGCTGAGCCTGCTTCTGGCGAGCCTGCTGATTATGTT
P21A-R	AACATAATCAGCAGGCTCGCCAGAAGCAGGCTCAGCAG
R33K-F	TTATTCCGAGCACCTTTAAACGCCCGGTGCTGAGCTGG
R33K-R	CCAGCTCAGCACCGGGCGTTTAAAGGTGCTCGGAATAA
L37S-F	ACCTTTCGCCGCCCGGTGTCAAGCTGGAAAGTGCAGAG
L37S-R	CTCTGCACTTTCCAGCTTGACACCGGGCGGCGAAAGGT
F66C-F	CTGCAGCCCGCTTCTGTGCAGCTTTGTGCCGGAAA
F66C-R	TTTCCGGCACAAAGCTGCACAGAAGCGGGCTGCAG
S83C-F	GATGCCGAAACAGACCTGCGTGAACAACTATGCGC
S83C-R	GCGCATAGTTGTTCACGCAGGTCTGTTTCGGCATC
linearized araC-ParaBAD-ID52-E-F	TGGTCTCATCCGCAGTTTGA
linearized araC-ParaBAD-ID52-E-R	TTTTTATAACCTCCTTAGAGCTCGA
α3-F	GCTCTAAGGAGGTTATAAAAAATGGAACGCTGGACCTTAC
α3-R	TCAAACTGCGGATGAGACCAACCGCCAGTACCCAGTTT
G4-F	GCTCTAAGGAGGTTATAAAAAATGGAACATTGGACTTTAAGC
G4-R	TCAAACTGCGGATGAGACCAACCGCCAGTACCCAGATCTT
mraY-F	TTAGTGGTGGTGGTGGTGGTGTAACCACACCTCGATGTAAAT

mraY-R	AAAGGAAAGGAGGAAAGAAATAATGCTTGAGCAAGTCATTCTGTT
linearized pLysS-pHLIP-F	TATTTCTTTCCTCCTTTCCTTTT
linearized pLysS-pHLIP-R	GGTTACACCACCACCACCACCACTAATT
SN20-F	CCTAGGTATAATACTAGTGTACCGGACCCTGGTCGGTTGTTTTAGAGCTAGAAATAGC
SN20-R	ACTAGTATTATACCTAGGACTGAGC
DH20-F	CCTAGGTATAATACTAGTCATTCCGACTCTGGAAGAGTGTTTTAGAGCTAGAAATAGC
DH20-R	ACTAGTATTATACCTAGGACTGAGC
slyD-up1000-F	GGCACCGAGTCGGTGCACAGTGATTTCATCCATATCTCC
slyD-up1000-R	TTCCCATGCTCAGGAGATATCTATCGAAAAGGTGACAAAAA
slyD-dn1000-F	TTTTTGTCACCTTTTCGATAGATATCTCCTGAGCATGGGAA
slyD-dn1000-R	TCGACTCTAGAGAATTCAAAAAAATCCGCATCAGGCGCA
dnaJ-up1000-F	GCACCGAGTCGGTGCAACCATGGGCGGTGTGATGA
dnaJ-up1000-R	AGGCTTTTGGGGAGGCTTTTTTGATTTGCCCCTAG
dnaJ-dn1000-F	GGCAAATCAAAAAAGCCTCCCCAAAAGCCTGCCCG
dnaJ-dn1000-R	GAGAATTCAAAAAAAAGAATGACCAGGCCAGTATA
Δ*slyD*-F	CGGGGATATCAGTGCCGTTAA
Δ*slyD*-R	AGACTTCACGCGAGCTTCGA
Δ*dnaJ*-F	CGTAAAGATGTTAACCCGGAC
Δ*dnaJ*-R	GATAGATAGCTGTTATCAAGGTACAC

Red letters indicate homology arms representing unrestricted cloning (RF-cloning) and polymerase chain reaction (PCR).

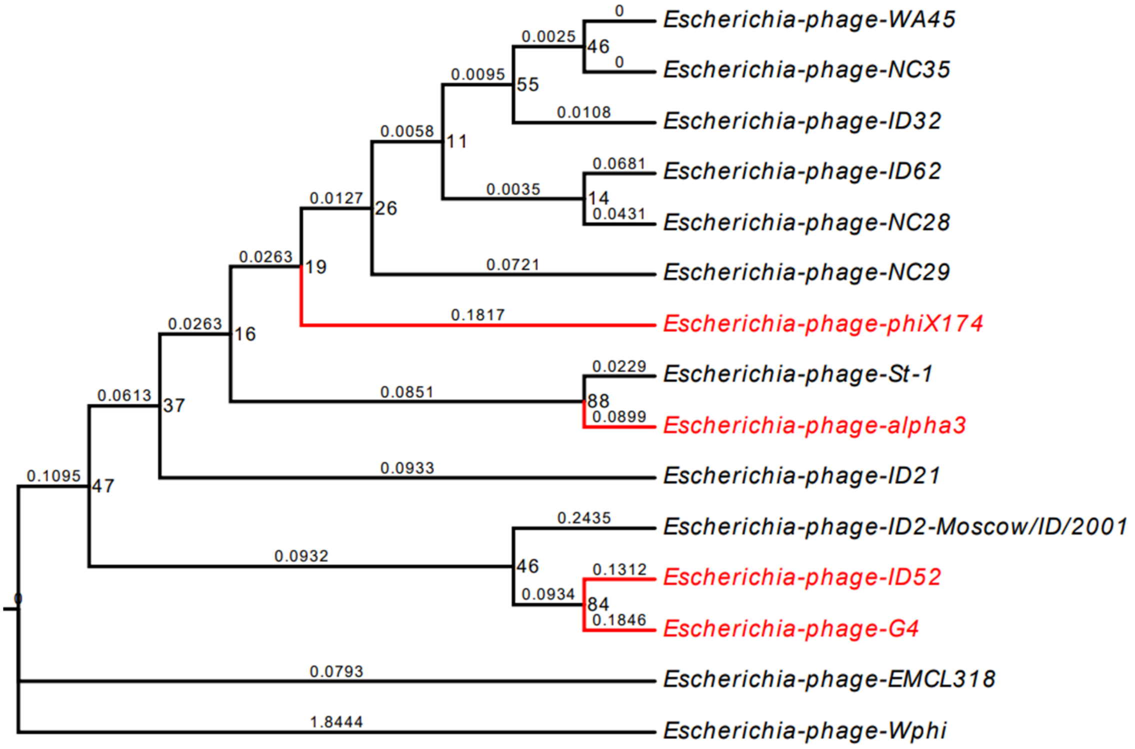

Figure S1. The evolutionary tree analysis of Enterobacteriaceae phage lysis gene E.

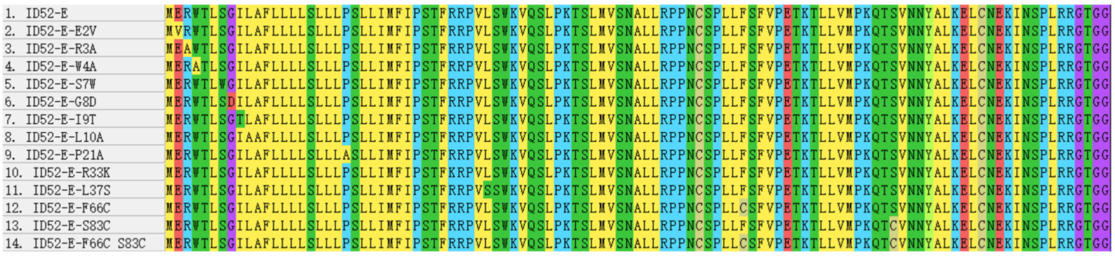

Figure S2. Comparison of the amino acid sequences of wild-type and mutant of phage ID52 lysis protein E.

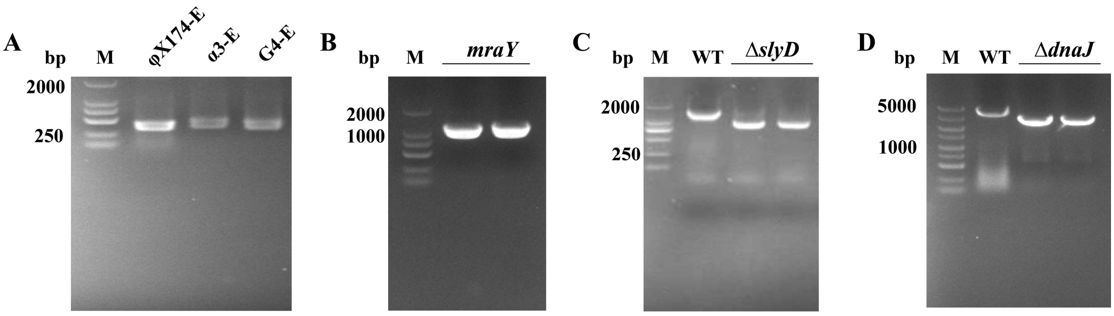

Figure S3. Colony analysis by agarose gel electrophoresis. (A) *E. coli* DH5α colonies containing plasmids araC-ParaBAD-φX174-E, araC-ParaBAD-G4-E, and araC-ParaBAD-α3-E, respectively. (B) *E. coli* DH5α colonies containing plasmids pLysS-*mraY*. (C) Colony PCR to identify the deletion of *slyD* gene in EcN. (D) Colony PCR to identify the deletion of *dnaJ* gene in EcN.

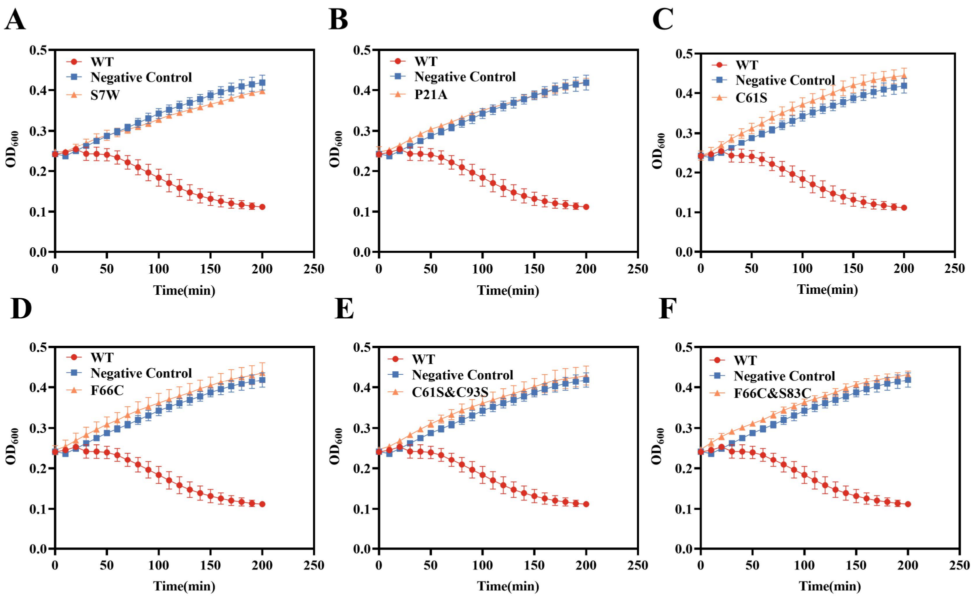

Figure S4. The lysis curves of EcN mutated to lose activity. The growth curves of at least five replicates of each mutant were monitored using OD_{600}.

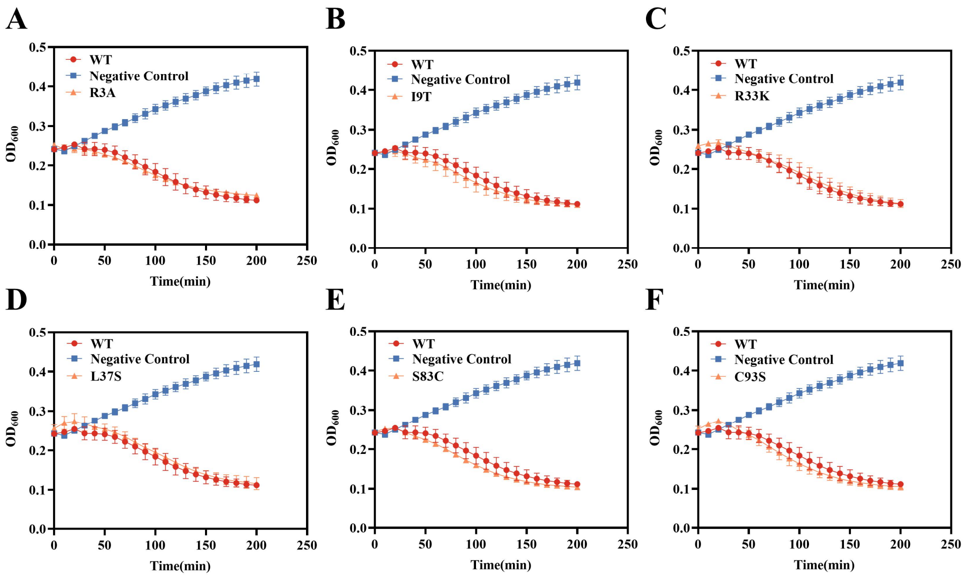

Figure S5. The lysis curves of the EcN mutant were not significantly affected. The growth curves of at least five replicates of each mutant were monitored using OD_{600}.

Table S3. Agricultural productivity and yield of two lysis proteins.

Lysis Protein E	Induction of OD600	Agricultural Productivity	Yield
ID52-E-W4A	6.0	60.39%	0.223 g/L
ID52-E-W4A	4.0	44.08%	0.185 g/L
φX174-E	4.0	3.09%	0.014 g/L

Western blot of the samples manifested that the lysis protein ID52-E-W4A could be expressed in EcN. The lysis protein ID52-E-W4A encoded 103 amino acids with the addition of the Strep-tag II tag and a molecular weight size of 12.93 kDa.

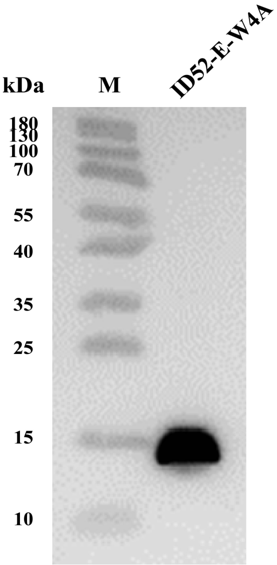

Figure S6. The expression of lysis protein ID52-E-W4A was verified by Western blot. M: 26616 prestained protein marker.

Table S4. The inactivated efficiency of different lysis protein E in EcN.

Initial Induction	Inactivated Efficiency (%)	
OD_{600} values	ID52-E	φX174-E
0.8	99.994	92.727
1.2	99.950	97.142
1.6	99.952	98.500
2.0	99.428	99.883
